# Alcohol and Energy Drink Use Among Adolescents and Young Adults: Insights from the ALIMA Study

**DOI:** 10.3390/nu18142323

**Published:** 2026-07-16

**Authors:** Federica Intorre, Maria Stella Foddai, Eugenia Venneria

**Affiliations:** Consiglio per la Ricerca in Agricoltura e l’Analisi dell’Economia Agraria—Council for Agricultural Research and Economics (CREA), Research Centre for Food and Nutrition (CREA—Food and Nutrition), Via di Vigna Murata, 605, 00143 Rome, Italy; mariastella.foddai@crea.gov.it (M.S.F.); eugenia.venneria@crea.gov.it (E.V.)

**Keywords:** adolescents, alcohol consumption, binge drinking, energy drinks, risk behaviors

## Abstract

Background/Objectives: Adolescence is a critical developmental stage marked by increased susceptibility to engaging in risky health behaviors, including the use of alcohol, energy drinks (EDs), and tobacco products. Methods: A cross-sectional sample of 1249 individuals aged 10–24 years was analyzed, stratified into two age groups (<16 and >16 years). The study sample comprised volunteers living in Rome, Italy, who were recruited for the ALIMA (ALImentazione Multiculturale negli Adolescenti) study. Categorical variables were analysed using the Pearson chi-square test and *p* < 0.05 was considered statistically significant. Results: Alcohol consumption was higher among participants aged ≥16 years (62.2%) than among those age <16 years (39.9%), without significant gender difference, with binge drinking also more common, especially among older males. ED use was widespread, more frequent in males, and often associated with alcohol consumption. Smoking prevalence differed by age group, reaching 41.5% among participants over 16 years of age, with higher rates among younger females. Finally, 17.3% reported combined use of alcohol, EDs, and tobacco, with significant gender differences in both age groups. Conclusions: These findings highlight a clear age-related escalation and co-occurrence of substance-use behaviors, with emerging gender differences in specific patterns. The results underscore the need for integrated, age-targeted prevention strategies addressing multiple substance use simultaneously to reduce long-term health risks and promote adolescent well-being.

## 1. Introduction

Adolescence represents a developmental period characterized by increased vulnerability to engaging in risky health behaviors, including the consumption of alcohol, caffeine and other stimulants, as well as tobacco products. The initiation of these substances during adolescence is influenced by complex interplay of psychological, social, and environmental factors, and is of public health concern worldwide due to their potent negative implications on physical, cognitive, and psychosocial development [1,2,3,4,5,6,7]. Sociodemographic determinants, peer influence, family environment, and targeted marketing strategies are primary drivers of adolescent substance-use behavior. Marketing efforts often capitalize on youth vulnerability by associating energy drinks and alcohol with increased social status, fun, and risk-taking, while tobacco marketing targets diverse adolescent subpopulations through product innovation and social media. This highlights the imperative for multidimensional research frameworks that incorporate neurobiological, psychosocial, and environmental factors to comprehensively address adolescent substance use [2,4,6].

Early alcohol consumption is consistently linked to a higher risk of developing alcohol-use disorders, impaired neurocognitive functioning, heightened risk-taking, and psychosocial maladjustment [8,9,10]. Alcohol dehydrogenase is the primary enzyme responsible for the oxidation of ethanol to acetaldehyde. Its activity develops progressively, beginning during the fetal stage and reaching stability in early adulthood. During adolescence, alcohol dehydrogenase function is generally comparable to that observed in adults; however, the rate of alcohol metabolism may vary due to ongoing hormonal changes. These variations can influence acetaldehyde production and individual sensitivity to alcohol, thereby affecting drinking behaviors and the risk of alcohol misuse during this developmental period [11,12].

Recent data show that alcohol consumption among Italian adolescents remains a major public health issue. In 2024, about 1.27 million people aged 11–24 were identified as at-risk drinkers, including about 580,000 minors. Binge drinking (defined in Italy as the consumption of six or more alcoholic drinks on a single occasion at least once in the past 12 months) [13] was observed in roughly 11% of young males and 6.9% of young females. The percentage of adolescent females engaging in binge drinking nearly doubled in the past decade [14].

Parallel to alcohol consumption, the popularity of energy drinks (EDs)—beverages high in caffeine, sugar, and other stimulants—has increased markedly among adolescents [15,16]. In Italy, ED consumption is highly prevalent among adolescents, with studies reporting that up to 57% have consumed EDs [17], consistent with more recent national surveys indicating lifetime prevalence rates above 60% [18]. This behavior appears less common among university students and is generally more frequent in males than females. [19,20]. Evidence suggests that high caffeine intake during this critical period is associated with adverse cardiovascular outcomes, disturbed sleep architecture, increased anxiety, and can potentiate risky behaviors [21,22].

A particular public health concern is the simultaneous consumption of EDs with other substances [23]. Of the 16-year-old students surveyed in Europe, 33.9% reported alcohol mixed with ED (AmED) use in the past year. The global prevalence observed among male students (37.3%) was higher than among female students (30.6%). In Italy the percentage of adolescents reporting AmED use is 26.2% [7]. The stimulant effects of caffeine mask alcohol’s sedating properties, leading to deceptive perceptions of sobriety, which may escalate alcohol intake and risky behaviors, including impaired driving and sexual risk-taking [21,24,25,26].

Tobacco use remains a leading preventable health risk [27], and adolescence is the most vulnerable stage for starting. The introduction of new nicotine products like e-cigarettes and heated tobacco has further complicated youth smoking trends. Exposure to nicotine during this developmental period can alter brain maturation—particularly areas controlling decision-making and reward—thus increasing the likelihood of addiction and polysubstance use [28].

In Italy, both cigarette smoking and e-cigarette use increase with age among adolescents. While prevalence is similar up to the age of 13, boys report slightly higher rates in the youngest group. From age 13 onwards, however, girls tend to smoke more than boys, and this difference widens with age. Overall, about 11% of adolescents report cigarette smoking and around 10% report e-cigarette use in the past 30 days [29].

This study offers a complementary perspective by investigating these behaviors simultaneously in a community-based sample of adolescents and young adults aged 10–24 years living in a large metropolitan setting. The broad age range allows the evaluation of critical developmental transitions from early adolescence to young adulthood. In addition, the study explores behavioral clustering and contextual patterns of substance use, including both alcohol and ED consumption during meals, and the concurrent use of alcohol, energy drinks, and tobacco products. By adopting an integrated approach, this study aims to provide a more comprehensive understanding of how multiple risk behaviors co-occur across developmental stages and genders, thereby generating evidence that may support more targeted and multifaceted prevention strategies. Addressing these intersecting substance-use behaviors is critical for reducing the burden of related health consequences and improving adolescent well-being on a global scale.

## 2. Materials and Methods

### 2.1. Participants and the Study Design

Volunteers were recruited between October 2020 and June 2025 from secondary schools and youth aggregation centres located in seven different municipalities, strategically distributed across the urban area of Rome. The sample included 1249 adolescents and young adults aged 10–24 years. The participating institutions were either affiliated with CREA—Food and Nutrition or voluntarily enrolled in the “Mappa della Città Educante 23–24 and 24–25” project promoted by Roma Capitale, for the implementation of the ALIMA (ALImentazione Multiculturale negli Adolescenti) study. The general purpose of the study is to assess whether and to what extent the interaction between different cultures and geographic backgrounds, within the context of globalization, has influenced adolescents’ lifestyles and dietary habits. After obtaining informed consent, trained interviewers administered the questionnaires in a single “face-to-face” assisted session. These instruments were designed to collect information on lifestyle, eating habits and food consumption. All data were collected anonymously to ensure confidentiality and processed in compliance with the European General Data Protection Regulation (GDPR 679/2016). The study was approved by the Territorial Ethics Committee “LAZIO AREA 2” (Registro Sperimentazione 248.24 CET2 CREA).

### 2.2. Lifestyle, Eating Habits and Food and Beverages Consumption

Information about demographic factors and social aspects (age, gender, country of origin, education and/or occupation, living arrangement), leisure-time activity (type and dedicated time), and eating habits (frequency of meal consumption, away-from-home eating, water, ED, soft drinks, and alcohol consumption -beer, wine, spirits-, eating differently from family members or roommates) were collected through a specifically designed lifestyle questionnaire. This instrument also captured contextual aspects of beverage consumption, such as their role during meals, social occasions, and eating outside home. Information was collected to characterize habitual intake and to identify their regular consumption [30,31,32].

Information on dietary habits was, moreover, collected using a qualitative Food Frequency Questionnaire (FFQ) designed to assess participants’ usual food and beverage consumption over the previous 12 months. For each food item, participants were asked to report their usual frequency of consumption by selecting one of several predefined categories, ranging from “never” to specific frequencies expressed as servings per day, per week, per month, or per year [30,31,32].

### 2.3. Statistical Analysis

All statistical analyses were performed using MedCalc Software (version 23.1.3 for Windows). Categorical variables were expressed as percentages, and associations between variables were assessed using the Pearson chi-square test. A *p*-Value of less than 0.05 was considered statistically significant.

## 3. Results

The sample included 1249 adolescents and young adults aged 10–24 years: 46.2% were <16 years, and 53.8% were >16 years (overall mean age 15.3 ± 2.0 years; males: 15.5 ± 2.1 years; females: 15.1 ± 1.9 years). The distribution of the population by gender shows variation across age groups (Table 1). Among individuals younger than 16 years, the proportion of males and females is nearly equal, accounting for 49.9% and 50.1%, respectively. In contrast, among individuals older than 16 years, males represent a higher proportion (58.3%), while females account for 41.7%. Overall, considering the total population, males constitute 54.4%, whereas females represent 45.6%.

Table 2 reports alcohol consumption patterns organized by age group without statistically significant differences between genders (*p*-Value = ns). Among individuals younger than 16 years, the majority are non-drinkers (60.1%), with 62.5% of males and 57.8% of females reporting no alcohol consumption. Conversely, 39.9% are drinkers, including 37.5% of males and 42.2% of females. In individuals older than 16 years, the pattern is reversed, with a higher proportion of drinkers (62.2%) compared to non-drinkers (37.8%). This trend is consistent across genders, with 63.0% of males and 61.1% of females reporting alcohol consumption. Regarding alcohol consumption during meals, no statistically significant gender differences are observed in individuals younger than 16 years (*p*-Value = ns). In this group, only a small proportion report drinking alcohol during meals (5.4%), including 6.2% of males and 4.5% of females. In contrast, among individuals older than 16 years, a statistically significant difference between genders is observed (*p*-Value < 0.001). Overall, 9.5% report consuming alcohol during meals; however, this behavior is more prevalent in males (13.8%) compared to females (3.6%). The majority in this age group report not consuming alcohol during meals (90.5%), particularly among females (96.4%) compared to males (86.2%). These findings suggest that alcohol consumption increases with age, while gender differences become more evident specifically in drinking habits during meals in individuals older than 16 years.

Table 3 presents the frequency of consumption of beer, wine, and spirits among individuals stratified by age group (<16 years and >16 years) and gender, along with associated statistical significance. In individuals younger than 16 years, no statistically significant differences are observed between males and females for any beverage type (*p*-Value = ns). The majority of this age group report never consuming alcohol, with 75.7% for beer, 68.6% for wine, and 74.5% for spirits. Monthly consumption is the next most common frequency. Daily consumption is rare across all beverage types, ranging from 0.2% to 0.5%. In individuals older than 16 years, a statistically significant difference is observed in beer consumption between genders (*p*-Value < 0.001), with males reporting higher frequencies of beer consumption. For wine and spirits, no significant gender differences are detected (*p*-Value = ns). In this age group, the most common frequency for wine and spirits is monthly (18.2% for spirits, 18.2% for wine), while daily consumption remains low (<1%). Among individuals >16 years, 57.7% report abstaining from beer, 55.1% from wine, and 47.3% from spirits, confirming an age-related increase in alcohol consumption.

Table 4 presents the prevalence of binge drinking among individuals stratified by age group (<16 years and >16 years) and gender. Among individuals younger than 16 years, there is no statistically significant difference between genders (*p*-Value = ns). In this group, as previously described, the majority are abstainers (60.1%), while 9.4% report binge drinking episodes. A part of the volunteers reports not knowing (6.9%) or not engaging in binge drinking (23.6%). In individuals older than 16 years, a statistically significant difference between genders is observed (*p*-Value = 0.004). Overall, 24.4% report binge drinking, with a higher prevalence among males (29.1%) than females (17.9%). Abstainers, also reported in Table 2, account for 37.8% of this age group, and those reporting “No” or “I don’t know” constitute 30.1% and 7.7%, respectively. These findings indicate that binge drinking increases with age and is more prevalent among males in the >16 years group, highlighting a significant age- and gender-related pattern in risky alcohol consumption behaviors.

Table 5 describes ED consumption, including intake during meals that differs significantly between genders in both age groups (<16 years, *p*-Value = 0.004; >16 years, *p*-Value < 0.001). Among individuals <16 years, 9.2% report consuming EDs during meals, with higher prevalence in males (12.5%) than females (5.9%), while 90.8% abstain. In those >16 years, 12.8% consume EDs during meals, again more frequently in males (18.1%) than females (5.4%). Regarding overall ED consumption, weekly intake is the most common frequency in both age groups. A substantial proportion never consumes EDs (50.4% for <16 years; 46.3% for >16 years). Males consistently report higher frequencies of consumption than females, indicating gender-related differences in both general and meal-associated ED intake. Although AmED consumption is lower in individuals under 16 years of age, the intake of spirits can reach up to 6 times per week (mean 2.2), and that of EDs up to 7 times per week (mean 2.2).

Table 6 summarizes smoking status among individuals stratified by age group (<16 years and >16 years) and gender. Among individuals younger than 16 years, there is a statistically significant difference between genders (*p*-Value < 0.001). A higher proportion of females are current smokers (30.8%) compared to males (14.2%), while 69.8% of the total population report never having smoked. Former smokers account for 7.6% of this age group. In individuals older than 16 years, no statistically significant gender differences are observed (*p*-Value = ns). Overall, 41.5% are current smokers, 7.9% are former smokers, and 50.6% report never having smoked. Current smoking is slightly more prevalent among females (46.4%) than males (38.0%), but the difference is not statistically significant. These data indicate that smoking prevalence increases with age.

Table 7 reiterates the three risk behaviours already extensively described in the previous tables together with the statistical analyses, and additionally includes their concurrent occurrence, namely alcohol consumption, energy drink use, and smoking. These simultaneous behaviors were reported by 13.3% of participants younger than 16 years and by 21.3% of those older than 16 years. Significant gender differences were observed in both age groups. Among younger adolescents, females were more likely than males to engage in all three behaviors simultaneously (18.3% vs. 9.4%, *p* < 0.001), whereas among older adolescents the opposite pattern was observed, with a higher prevalence among males (24.7% vs. 16.4%, *p*-Value = 0.009).

## 4. Discussion

This research provides a comprehensive overview of substance-related behaviors (alcohol, caffeine and other stimulants, as well as tobacco) among adolescents and young adults enrolled in the ALIMA study, highlighting consistent age-related increases in consumption, alongside gender-specific patterns. Overall, the results are largely coherent with evidence from major European surveillance systems, particularly Health Behaviour in School-aged Children (HBSC) [33] and European School Survey Project on Alcohol and Other Drugs (ESPAD) [18], both of which provide robust and comparable population-based data, underscoring some critical public health concerns. The age threshold of 16 years was selected because it represents a critical developmental transition during which autonomy, peer influence, social activities, and exposure to substance use tend to increase substantially. Moreover, this cutoff is consistent with the age range commonly investigated in European surveillance systems such as ESPAD [18], facilitating comparisons with existing literature. The division also allowed us to evaluate whether the prevalence and clustering of alcohol, energy drink, and tobacco use differed before and after this key developmental stage, characterized by important psychological, social, and behavioral changes.

Across all behaviors examined, age emerges as the strongest determinant of substance-use prevalence and frequency. Alcohol consumption, binge drinking, ED use, and smoking are consistently less prevalent among individuals younger than 16 years and increase markedly in those older than 16. This pattern closely mirrors international data indicating that early adolescence is characterized mainly by experimentation, whereas mid to late adolescence marks a transition toward more frequent and risk-prone behaviors [18,33,34].

In our study, among individuals younger than 16 years, the majority were non-drinkers (60.1%), with no statistically significant gender differences. This finding aligns with several European studies showing that, in early adolescence, alcohol use prevalence is relatively low, and gender differences are minimal or absent. At this stage, alcohol consumption appears to be more strongly influenced by family environment, parental supervision, and school-related protective factors rather than by consolidated gender norms. In contrast, among individuals older than 16 years, the proportion of drinkers exceeds that of non-drinkers (62.2% vs. 37.8%), with similar prevalence rates in males and females. This lack of gender difference reflects the well-documented convergence in alcohol use between males and females during adolescence [35].

However, gender-specific differences emerge when considering the context of alcohol consumption. Drinking during meals was rare among those younger than 16 (5.4%) and did not differ by gender, suggesting that alcohol use at this stage is mainly sporadic and not yet integrated into structured social or family contexts. In contrast, among individuals over 16, alcohol consumption during meals showed a clear gender disparity, with higher prevalence among males (13.8% vs. 3.6%). This observation is consistent with evidence indicating that, in later adolescence, sociocultural norms increasingly shape drinking patterns, with males more likely to incorporate alcohol into routine social practices and females generally adopting more restrained behaviors [36,37].

In Mediterranean settings, alcohol consumption during meals is culturally normalized, but in adolescence it may normalize alcohol use early, especially among males. Family meals tend to protect females, while males are more influenced by peers, increasing alcohol use in social settings [38,39,40]. Over the past 10 years, the trend of alcohol consumption outside meals has consistently increased for both genders, confirming a progressive shift away from the traditional Mediterranean drinking pattern. In our sample, only a small proportion of individuals consume alcohol during meals. These findings are consistent with the trend observed in Italy: notably, 28,6% of males and 25,1% of females aged 11–24 report consuming alcohol outside meals [14].

With respect to beverage types, beer consumption is more common among males, whereas wine and spirits show more similar patterns between genders. This likely reflects gender-specific social norms and drinking contexts. From a public health perspective, the higher prevalence of beer consumption after age 16 is particularly relevant, as it is associated with greater total alcohol intake and an increased risk of heavy episodic drinking [14,18,41].

In our study, binge drinking prevalence increases markedly with age and shows clearer gender differences in late adolescence, with males at higher risk. These findings are consistent with both European and Italian surveillance data [14,18,33]. While gender differences are less pronounced in early adolescence—with some studies reporting similar or even higher prevalence among females by age 15 [42]—they become more evident later, reflecting the progressive influence of gender-specific social norms. In Italy, binge drinking affects 11.1% of males and 6.9% of females aged 11–24 years, with a marked increase from early to late adolescence [14].

The persistence of a substantial proportion of abstainers even among older adolescents (37.8%) is noteworthy and aligns with recent evidence suggesting increasing polarization in youth drinking behaviors. While a subgroup engages in risky patterns such as binge drinking, another remains abstinent, reflecting broader trends toward delayed initiation or reduced alcohol use [18].

From a public health perspective, the gender disparity observed in the >16 years group is particularly relevant. Numerous studies have demonstrated that binge drinking is more strongly associated with acute harms and long-term adverse outcomes, especially among males. Neurodevelopmental research further highlights that binge drinking during adolescence may have lasting effects on brain structure and function, given the ongoing maturation of neural systems involved in executive control and reward processing [43].

Our findings confirm that ED consumption increases with age, is more frequent among males, and is not limited to occasional use, with a proportion of adolescents consuming EDs weekly and during meals, consistent with patterns reported in the European adolescent population [21,44]. In our study, ED consumption during meals was reported by 9.2% of individuals under 16 and increased to 12.8% among those older than 16, with consistently higher prevalence among males. Although consumption in a meal context has been less extensively studied, it may represent a marker of habitual use rather than sporadic intake. Moreover, adolescents under 16 years of age tend to consume EDs primarily as standalone beverages, whereas individuals older than 16 years are more likely to associate their consumption with alcohol intake, particularly in the form of mixed drinks.

Previous research suggests that ED consumption is often embedded in daily routines (e.g., school, physical activity, or screen time), and that males tend to report higher consumption across both frequency and context, possibly reflecting marketing strategies and greater sensation-seeking behaviors [1,45].

The observed gender differences in ED use are consistent with broader epidemiological evidence linking ED consumption to other risk behaviors, including alcohol use, binge drinking, smoking, and unhealthy dietary habits. Frequent ED consumers are more likely to engage in multiple risk behaviors, indicating overlapping behavioral profiles [1,22]. The coexistence of repeated ED and alcohol intake in older adolescents further raises concerns about cumulative exposure to stimulants and psychoactive substances during a critical developmental period [22].

Tobacco use shows patterns consistent with European surveillance data, with prevalence increasing with age and gender differences evolving over adolescence [18,33]. Notably, our findings indicate higher smoking prevalence among females in individuals younger than 16 years, in line with evidence that adolescent girls in several European countries are reaching or exceeding boys in smoking rates by mid adolescence [18,42,46]. This trend has been attributed to sociocultural factors, gender-specific beliefs, and targeted marketing strategies. In older adolescents, smoking prevalence converges between genders, reflecting the broader European trend toward diminishing gender differences in tobacco use [18,47,48].

Although many of the prevalence estimates reported in the present study are consistent with findings from European surveillance systems (HBSC [33] and ESPAD [18]), the added value of the ALIMA study lies in its integrated analytical framework. Rather than examining individual behaviors separately, the present study simultaneously evaluates alcohol consumption, binge drinking, energy drink use, and smoking within the same population, allowing the characterization of their co-occurrence and clustering. Moreover, in this study there is the inclusion of participants spanning a broad developmental period (10–24 years), which enables the identification of age-related transitions that are not fully captured by surveillance systems focused on narrower age groups. This approach highlights how the escalation of substance use behaviors occurs progressively across adolescence and young adulthood and how gender differences emerge differently according to the specific behavior examined. These findings confirm that the three risk behaviours do not occur in isolation but tend to co-occur, highlighting a pattern of behavioural clustering among adolescents. The inclusion of concurrent alcohol consumption, energy drink use, and smoking underscores the potential synergistic effect of these behaviours, which may reflect shared underlying determinants such as risk-taking propensity and social influences. This pattern further emphasizes the need for integrated prevention strategies addressing multiple behaviours simultaneously rather than in isolation. These aspects are less frequently explored in large surveillance studies and may contribute to a better understanding of behavioral trajectories and opportunities for integrated prevention.

## 5. Limitations and Strengths

Some limitations should be acknowledged. Its cross-sectional design does not allow causal inference, and the use of self-reported data may introduce recall and social desirability bias, particularly for sensitive behaviors such as alcohol consumption, binge drinking, smoking, and ED use. The study was designed to provide a descriptive assessment of the prevalence and clustering of risk behaviors. Given its cross-sectional design and the absence of a predefined etiological framework for predictor selection, multivariable regression analyses were not conducted, as the primary aim was not to identify independent determinants but rather to describe behavioral patterns across demographic groups. Additionally, another limitation is the use of convenience sampling. Participants were recruited on a voluntary basis from schools and youth centres involved in the ALIMA project; therefore, although comparable in age to large European surveys, the sample may not be fully representative of the general adolescent population, limiting the generalizability of the findings. Furthermore, data collection was conducted over a relatively long period (2020–2025). During these years, significant social and environmental changes occurred, including the COVID-19 pandemic and the subsequent resumption of social activities, which may have influenced substance use behaviours among adolescents. Patterns of alcohol consumption, energy drink use, and smoking may have changed over time because of evolving social norms, availability of products, public health policies, and post-pandemic behavioural adaptations. As the present analyses were not designed to evaluate temporal trends, potential period effects could not be formally assessed and may have contributed to variability in the observed prevalence estimates. Nevertheless, the primary objective of the study was to describe overall patterns, co-occurrence, and age- and gender-related differences in substance use behaviours within the ALIMA cohort rather than to monitor trends over time.

Despite these limitations, the present study is strengthened by several methodological and conceptual features that enhance the reliability and relevance of its findings. First, the relatively large sample size and the inclusion of a broad age range provide sufficient statistical power and allow for meaningful comparisons across key developmental stages. The stratification into two age groups (<16 and >16 years) is particularly valuable, as it captures critical transitions within adolescence and early adulthood, enabling the identification of age-related trends in substance. Furthermore, the study adopts a comprehensive approach by simultaneously examining alcohol, ED, and tobacco use, offering an integrated perspective on adolescent risk behaviors. The detailed assessment of consumption patterns, including frequency, context of use (e.g., during meals), and specific behaviors such as binge drinking and AmED, further enriches the analysis. In addition, the inclusion of gender-disaggregated data allows the identification of specific differences, which are essential for the development of targeted prevention strategies.

## 6. Conclusions

Overall, the study identifies a convergent pattern of multiple risk behaviors that increase with age and exhibit gender-specific clustering. The co-occurrence of alcohol use, binge drinking, energy drink (ED) consumption, and smoking—particularly among older adolescents—reflects the polysubstance use profiles widely reported in European populations. The findings should be interpreted as evidence of behavioral associations and clustering rather than causal relationships. Due to the cross-sectional design, no conclusions can be drawn regarding the directionality of the observed associations between alcohol consumption, smoking, and ED use. The study was primarily intended to describe the prevalence and co-occurrence of these risk behaviors, providing an epidemiological overview rather than identifying causal mechanisms or independent predictors.

Overall, these findings highlight the need for early, integrated, and age-appropriate prevention strategies that are also gender-sensitive. Such approaches should address not only individual substances but also the broader lifestyle and social contexts in which these behaviors occur, with the aim of promoting healthier habits and reducing risk exposure. Particular attention should be given to early smoking initiation among girls, the higher prevalence of heavy episodic drinking among boys, and the widespread accessibility and normalization of ED consumption among adolescents. In this context, targeted educational interventions are essential to prevent the early adoption of risky behaviors that may persist into adulthood. School-based smoking prevention programs should be introduced before the age of 14 years, ideally during lower secondary school, focused on the development of critical thinking, risk consciousness, and self-regulation skills. Alongside educational campaigns, policymakers should strengthen the enforcement of existing minimum legal drinking-age regulations and improve age-verification measures in both retail and social settings and consider stricter regulations on the marketing and sale of energy drinks to minors. Moreover, integrating education on diet quality with awareness of substance-related risks may help adolescents make more informed choices, reduce the clustering of unhealthy behaviors, and foster the development of long-term health-promoting lifestyles.

## Figures and Tables

**Table 1:** Distribution of the population by gender and age.

	<16 Years	>16 Years	Total
	**N (%)**	**N (%)**	**N (%)**
**Males**	288 (49.9)	392 (58.3)	680 (54.4)
**Females**	289 (50.1)	280 (41.7)	569 (45.6)

**Table 2:** Alcohol consumption and intake during meals by gender and age.

Alcohol Consumption	<16 Years (*p*-Value = ns)			>16 Years (*p*-Value = ns)		
	**Males** **N (%)**	**Females** **N (%)**	**Total** **N (%)**	**Males** **N (%)**	**Females** **N (%)**	**Total** **N (%)**
**Teetotallers**	180 (62.5)	167 (57.8)	347 (60.1)	145 (37.0)	109 (38.9)	254 (37.8)
**Drinkers**	108 (37.5)	122 (42.2)	230 (39.9)	247 (63.0)	171 (61.1)	418 (62.2)
**Alcohol** **during meals**	**<16 years** **(*p*-Value = ns)**			**>16 years** **(*p*-Value < 0.001)**		
	**Males** **N (%)**	**Females** **N (%)**	**Total** **N (%)**	**Males** **N (%)**	**Females** **N (%)**	**Total** **N (%)**
**Yes**	18 (6.2)	13 (4.5)	31 (5.4)	54 (13.8)	10 (3.6)	64 (9.5)
**No**	270 (93.8)	276 (95.5)	546 (94.6)	338 (86.2)	270 (96.4)	608 (90.5)

Statistical analysis: Chi square test; ns = not significant.

**Table 3:** Frequency of consumption by type of alcoholic beverage and by gender and age.

	<16 Years								
Frequency	Beer (*p*-Value = ns)			Wine (*p*-Value = ns)			Spirits (*p*-Value = ns)		
	**Males** **N (%)**	**Females** **N (%)**	**Total** **N (%)**	**Males** **N (%)**	**Females** **N (%)**	**Total** **N (%)**	**Males** **N (%)**	**Females** **N (%)**	**Total** **N (%)**
**Daily**	3 (1.0)	0 (0.0)	3 (0.5)	1 (0.3)	1 (0.3)	2 (0.3)	0 (0.0)	1 (0.3)	1 (0.2)
**Weekly**	22 (7.69)	17 (5.9)	39 (6.8)	15 (5.2)	10 (3.5)	25 (4.3)	19 (6.6)	19 (6.6)	38 (6.6)
**Monthly**	29 (10.1)	27 (9.3)	46 (9.7)	37 (12.8)	40 (13.8)	77 (13.3)	27 (9.4)	40 (13.8)	67 (11.6)
**Yearly**	20 (6.9)	22 (7.6)	42 (7.3)	33 (11.5)	44 (15.2)	77 (13.3)	24 (8.3)	17 (5.99)	41 (7.1)
**Never**	214 (74.3)	223 (77.2)	437 (75.7)	202 (70.1)	194 (67.1)	396 (68.6)	218 (75.7)	212 (73.4)	430 (74.5)
	**>16 years**								
**Frequency**	**Beer** **(*p*-Value < 0.001)**			**Wine** **(*p*-Value = ns)**			**Spirits** **(*p*-Value = ns)**		
	**Males** **N (%)**	**Females** **N (%)**	**Total** **N (%)**	**Males** **N (%)**	**Females** **N (%)**	**Total** **N (%)**	**Males** **N (%)**	**Females** **N (%)**	**Total** **N (%)**
**Daily**	7 (1.8)	1 (0.4)	8 (1.2)	4 (1.0)	1 (0.4)	5 (0.7)	4 (1.0)	0 (0.0)	4 (0.6)
**Weekly**	67 (17.1)	26 (9.3)	93 (13.8)	43 (11.0)	16 (5.7)	59 (8.8)	69 (17.6)	51 (18.2)	120 (17.9)
**Monthly**	69 (17.6)	35 (12.5)	104 (15.5)	66 (16.8)	56 (20.0)	122 (18.2)	65 (16.6)	67 (20.4)	122 (18.2)
**Yearly**	49 (12.5)	30 (10.7)	79 (11.8)	69 (17.6)	47 (16.8)	116 (17.3)	60 (15.3)	48 (17.1)	108 (16.1)
**Never**	200 (51.0)	188 (67.1)	388 (57.7)	210 (53.6)	160 (57.1)	370 (55.1)	194 (49.5)	124 (44.3)	318 (47.3)

Statistical analysis: Chi square test; ns = not significant.

**Table 4:** Prevalence of binge drinking by gender and age.

Binge Drinking	<16 Years (*p*-Value = ns)			>16 Years (*p*-Value = 0.004)		
	**Males** **N (%)**	**Females** **N (%)**	**Total** **N (%)**	**Males** **N (%)**	**Females** **N (%)**	**Total** **N (%)**
**No**	65 (22.6)	71 (24.6)	136 (23.6)	108 (27.6)	94 (33.6)	202 (30.1)
**Yes**	26 (9.0)	28 (9.7)	54 (9.4)	114 (29.1)	50 (17.9)	164 (24.4)
**I don’t know**	17 (5.9)	23 (8.0)	40 (6.9)	25 (6.4)	27 (9.6)	52 (7.7)
**Teetotaller**	180 (62.5)	167 (57.8)	347 (60.1)	145 (37.0)	108 (38.9)	253 (37.8)

Statistical analysis: Chi square test; ns = not significant.

**Table 5:** Energy drink (ED) consumption and intake during meals by gender and age.

ED During Meals	<16 Years (*p*-Value = 0.004)			>16 Years (*p*-Value < 0.001)		
	**Males** **N (%)**	**Females** **N (%)**	**Total** **N (%)**	**Males** **N (%)**	**Females** **N (%)**	**Total** **N (%)**
**Yes**	36 (12.5)	17 (5.9)	53 (9.2)	71 (18.1)	15 (5.4)	86 (12.8)
**No**	252 (87.5)	272 (94.1)	524 (90.8)	321 (81.9)	265 (94.6)	586 (87.2)
**ED** **consumption**	**<16 years** **(*p*-Value = 0.001)**			**>16 years** **(*p*-Value < 0.001)**		
	**Males** **N (%)**	**Females** **N (%)**	**Total** **N (%)**	**Males** **N (%)**	**Females** **N (%)**	**Total** **N (%)**
**Daily**	17 (5.9)	16 (5.5)	33 (5.7)	32 (8.2)	9 (3.2)	41 (6.1)
**Weekly**	78 (27.1)	39 (13.5)	117 (20.3)	134 (34.2)	34 (12.1)	168 (25.0)
**Monthly**	46 (16.0)	47 (16.3)	93 (16.1)	69 (17.6)	27 (9.6)	96 (14.3)
**Yearly**	21 (7.3)	22 (7.6)	43 (7.5)	29 (7.4)	27 (9.6)	56 (8.3)
**Never**	126 (43.8)	165 (57.1)	291 (50.4)	128 (32.7)	183 (65.4)	311 (46.3)

Statistical analysis: Chi square test.

**Table 6:** Smoking status by gender and age.

Smoking	<16 Years (*p*-Value < 0.001)			>16 Years (*p*-Value = ns)		
	**Males** **N (%)**	**Females** **N (%)**	**Total** **N (%)**	**Males** **N (%)**	**Females** **N (%)**	**Total** **N (%)**
**Current smokers**	41 (14.2)	89 (30.8)	130 (22.5)	149 (38.0)	130 (46.4)	279 (41.5)
**Former smokers**	16 (5.6)	28 (9.7)	44 (7.6)	31 (7.9)	22 (7.9)	53 (7.9)
**Never smokers**	231 (80.2)	172 (59.5)	403 (69.8)	212 (54.1)	128 (45.7)	340 (50.6)

Statistical analysis: Chi square test; ns = not significant.

**Table 7:** Behavioural clustering (Alcohol consumption, Energy Drink use, and smoking habits) by gender and age.

	<16 Years			>16 Years		
	**Males** **N (%)**	**Females** **N (%)**	**Total** **N (%)**	**Males** **N (%)**	**Females** **N (%)**	**Total** **N (%)**
**Drinkers**	108 (37.5)	122 (42.2)	230 (39.9)	247 (63.0)	171 (61.1)	418 (62.2)
**ED consumers**	162 (56.2)	124 (42.9)	286 (49.6)	264 (67.3)	97 (34.6)	361 (53.7)
**Smokers**	41 (14.2)	89 (30.8)	130 (22.5)	149 (38.0)	130 (46.4)	279 (41.5)
	**<16 years**			**>16 years**		
	(***p*-Value < 0.001**)			(***p*-Value = 0.009**)		
**Behavioural clustering**	24 (9.4)	53 (18.3)	77 (13.3)	97 (24.7)	46 (16.4)	143 (21.3)

Statistical analysis: Chi square test.

## Data Availability

The archived data and all the elaboration and analysis generated and used for the presentation of results in this study are fully available upon request due to privacy and ethical restrictions from the corresponding author.
